# Diffuse panbronchiolitis in a patient with common variable immunodeficiency: a casual association or a pathogenetic correlation?

**DOI:** 10.1186/1746-1596-9-12

**Published:** 2014-01-20

**Authors:** Alessandro Ginori, Aurora Barone, David Bennett, Marie Aimée Gloria Munezero Butorano, Maria Grazia Mastrogiulio, Antonella Fossi, Paola Rottoli, Donatella Spina

**Affiliations:** 1 Department of Medical Biotechnologies, Pathology Unit, University of Siena, strada delle Scotte 6, Siena 53100, Italy; 2 Respiratory Diseases and Lung Transplantation Unit, Regional Center for Sarcoidosis and other Interstitial Lung Diseases, University of Siena, viale Bracci 16, Siena 53100, Italy

**Keywords:** Diffuse panbronchiolitis, Common variable immunodeficiency, Foamy macrophages, Lymphocytes, Interstitial lung disease

## Abstract

Diffuse panbronchiolitis (DPB) is an idiopathic inflammatory disease that seems to have an immunological pathogenesis and that causes a severe progressive suppurative and obstructive respiratory disorder. Common variable immunodeficiency (CVID) is the most common serious primary immunodeficiency and it is often associated with respiratory diseases. Herein, we describe a case of DPB in a 41-year-old man affected by CVID. We examined the patient’s lungs, focusing on the characteristics of the inflammatory cells and of the foamy macrophagic nodules typical of DPB. Immunohistochemical typing of the lymphocytic infiltrate showed that B-cells were almost absent, matching the immunological profile of CVID. The case described is the first case reported in the literature of DPB in a patient affected by CVID. Moreover it seems to confirm the correlation between an immunodeficiency status and the development of DPB and provides more information on the accumulation of nodules of foamy macrophages in DPB.

**Virtual slides:**

The virtual slide(s) for this article can be found here: http://www.diagnosticpathology.diagnomx.eu/vs/5310709471138338.

## Background

Diffuse panbronchiolitis (DPB) is an idiopathic inflammatory disease that is characterized by progressive suppurative and obstructive airway disease, which, if left untreated, gives rise to bronchiectases, respiratory failure and death [1]. Chronic pulmonary obstructive disease (COPD) is characterized by irreversible airflow limitation and abnormal inflammatory response to noxious gases, especially cigarette smoke [2]. Many pathogenetic mechanisms seem to be involved in the development of COPD, including delayed apoptosis of neutrophils [2], aberrant methylation of tumor suppressor genes [3] and an immune-modulatory role of surfactant proteins such as pulmonary haptoglobin [4]. DPB was proposed as a disease entity, distinct from COPD, by Homma and Yamanaka in the 1960s in Japan, but it was not internationally accepted until the 1980s [5]. The term “diffuse” refers to the distribution of the lesions throughout both lungs, and the term “pan-” refers to the inflammation which involves all the layers of the respiratory bronchioles [6]. Outside Asia, only a limited number of cases have been reported. The prevalence of clinician-diagnosed DPB is 11 cases per 100,000 people [7], with a male to female ratio of 1.4 to 1 and a median age of 40-60 years. Patients suffer from chronic bacterial infection due to *Haemophilus influenzae*, *Streptococcus pneumoniae* and other species in the airways. Bacteria in the airway are gradually replaced by *Pseudomonas aeruginosa* and the disease is progressive in nature [8]. The prognosis of patients with DPB was poor, with 5- and 10-year survival rates of 62.1 and 33.2%, respectively. However, long-term treatment with erythromycin has increased the 10-year survival rate to >90% [6]. Although nothing is known about the aetiology of this disease, the finding of DPB among East Asians, including Asian emigrants, led to the suggestion that there could be a major susceptibility gene for DPB, located between the Human Leukocyte Antigen (HLA)-A and HLA-B loci [8]. Moreover, DPB has also been compared and associated with cystic fibrosis (CF), bare lymphocyte syndrome (BLS) type I, human T-cell lymphotropic virus type 1 (HTLV-1) and rheumatoid arthritis [6,9]. Common variable immunodeficiency (CVID) is the most common severe primary immunodeficiency with a reported prevalence of 1:30,000. CVID is a diagnosis of exclusion based on the presence of hypogammaglobulinaemia of at least two immunoglobulin isotypes, recurrent sinopulmonary infections, and impaired functional antibody responses, that include absent isohaemagglutinins, poor responses to protein or polysaccharide vaccines, or both [10]. The cellular characteristics of the immune system in CVID are complex with several numerical and functional defects involving both B and T lymphocytes, natural killer cells, macrophages and monocytes. In patients with CVID, respiratory diseases are a significant cause of morbidity and mortality. Histologic studies revealed that interstitial lung disease (ILD) in the context of CVID may manifest as sarcoid-like granuloma, organizing pneumonia, nonspecific interstitial pneumonia (NSIP), follicular bronchiolitis and lymphocytic interstitial pneumonia (LIP). These different patterns may be found in adjacent zones or even intermingled within one lung specimen [11]. In particular, LIP and follicular bronchiolitis belong to the same spectrum of benign lymphoproliferative disorders of the lungs [12] and often co-exist in CVID with pulmonary granulomatous diseases. For this reason they have recently been grouped together under the term granulomatous lymphocytic interstitial lung disease (GLILD) [13]. Herein, we present a case of DPB in a man affected by CVID, and a pathogenetic correlation between these two entities is hypothesized. To the best of our knowledge, the association between DPB and CVID has never been described in the literature.

## Case presentation

### Clinical summary

**Figure 1:**
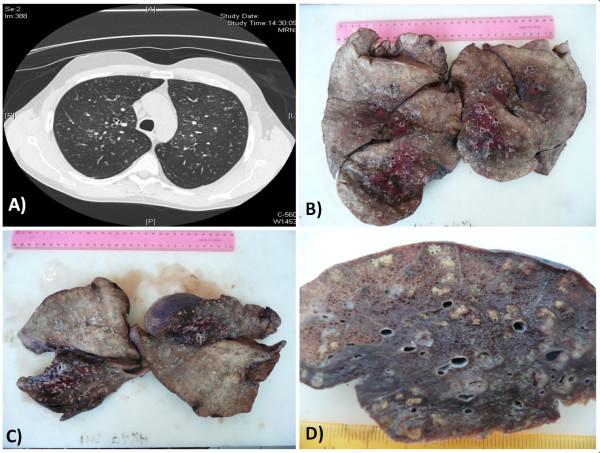
**Chest HRCT scan and gross examination of the lungs. (A)** HRCT scan: nodular shadows were distributed in a centrilobular fashion, often extending to small branching linear areas of attenuation (“tree-in-bud” pattern); **(B-D)** gross examination of the lungs showed bilateral multiple white-yellowish small nodules centred on small airways.

A 41-year-old Caucasian man, never smoker, had been referred to the Respiratory Diseases and Lung Transplantation Unit of our Hospital to be evaluated for lung transplantation because of chronic respiratory failure secondary to chronic lung disease and CVID. Diagnosis of CVID had been made when the patient was 22 years old for recurrent respiratory tract infections; he had a complete deficiency of the production of all immunoglobulin classes (IgA, IgM, IgG and IgE). He worked in textile industry until the age of 38 when he developed chronic respiratory failure and started oxygen-therapy. At 33 years old he had also been diagnosed with undifferentiated spondyloarthritis, treated with low dose of steroids. Chronic infection of respiratory tract by *Haemophilus influenzae* was present from the age of 24 and fifteen years later also *Streptococcus pneumoniae* was isolated in his sputum. When the patient came to our observation, he was on 24 h oxygen-therapy for type I chronic respiratory failure (blood gas analysis showed pH 7,40, pO2 66 mmHg, pCO2 38 mmHg, HCO3- 22 mmol/L with O2-therapy 2 L/min with nasal cannula). He was in New York Heart Association (NYHA) functional class III and pulmonary function tests showed a very severe obstructive respiratory deficiency with marked increase of static pulmonary volumes (FVC: 1180 ml, 25,2%; FEV1: 540 ml, 13,9%; FEV1/FVC ratio: 45%, RV: 9360 ml, 482%; TLC: 11170 ml, 161%). At chest auscultation bilateral crackles and wheezes were present. All the microbiologic examinations and PCR detection of most common respiratory viruses (including HTLV-1) were negative. A chest High Resolution Computed Tomography (HRCT)-scan showed mild bilateral dilatation of airways, bronchial wall thickening and a centrilobular distribution of nodular shadows, often extending to small, branching linear areas of attenuation (“tree-in-bud” pattern), predominantly in the middle and upper fields, consistent with bronchiolitis (Figure 1A). Panlobular emphysema was present in the lower lobes. Interestingly, HRCT alterations were moderate in contrast to respiratory impairment as documented by clinical and functional findings. Peripheral immunophenotype showed increased circulating T lymphocytes (94%) with conserved CD4/CD8 ratio, reduction of NK lymphocytes (3%) and total absence of B-lymphocytes. Serum IgA, IgM, IgG and IgE were undetectable. HLA typing showed positivity for HLA-A*01, HLA-A*11, HLA-B*51, HLA-B*52, DRB1*12 and DRB1*15. The patient was listed for lung transplantation and 8 months later he underwent bilateral lung transplantation.

### Pathologic findings

**Figure 2:**
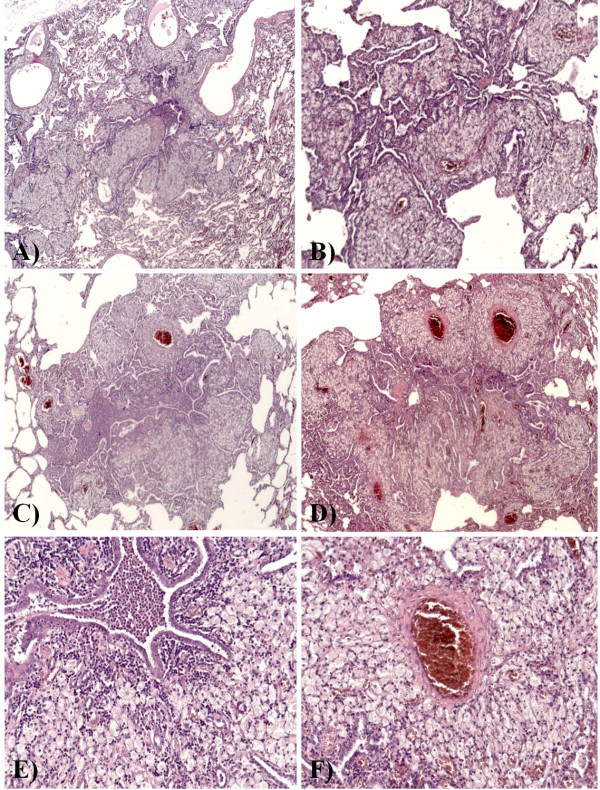
**Histological features. (A-D)** Transmural and peribronchial infiltration by lymphoctytes and histiocytes was found, with prominent and diffuse involvement of respiratory and terminal bronchioles; most of the histiocytes were foamy macrophages, which formed “nodules”, distributed especially in the wall of respiratory bronchioles, in the surrounding interalveolar septa and around the blood and lymphatic vessels. HE, x25. **(E)** The inflammatory infiltrate showed a characteristic topography: the bronchiolar lumen contained neutrophils, while the peribronchiolar infiltrate was constituted of an inner layer of lymphocytes and an outer layer of histiocytes. HE, x200. **(F)** Note the exuberant perivascular distribution of foamy macrophages. HE, x200.

**Figure 3:**
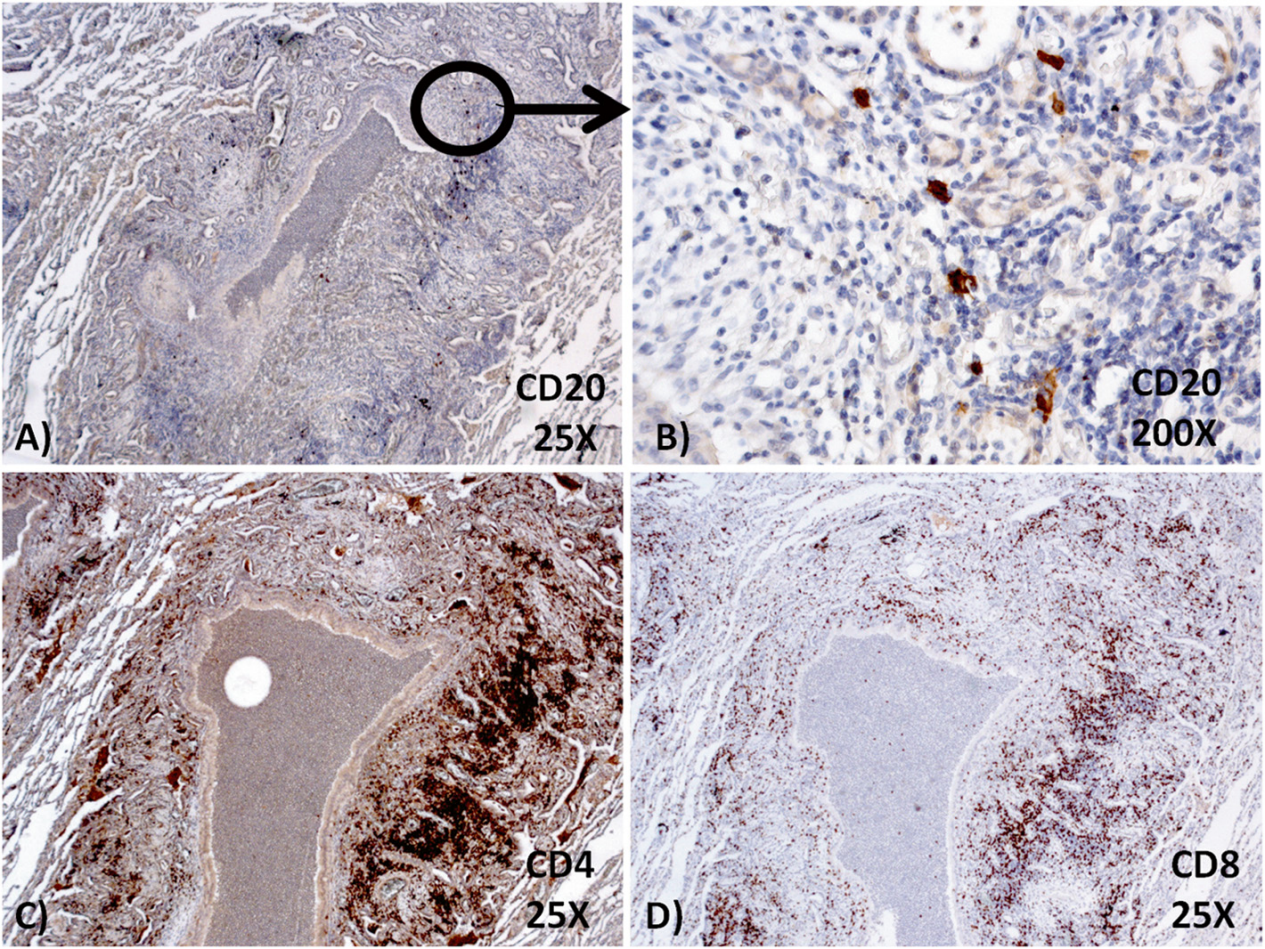
**Immunohistochemical stains. (A-B)** CD20+ B-cells were almost absent in the lymphocytic infiltrate; **(C-D)** a heterogeneous population of CD4+ and CD8+ T-cells was present.

Lung tissue was fixed in 10% neutral buffered formalin. At gross examination, the lungs showed multiple bilateral white-yellowish small nodules, 2-5 mm in diameter, centred on small airways, more frequently in the upper lobes, associated with bronchiectases (Figure 1B-D). Representative samples were taken and paraffin embedded. Four micrometer-thick sections were stained with haematoxylin and eosin (HE) and examined by light microscopy. Histologically, transmural and peribronchial infiltration by lymphoctytes and histiocytes was found, with prominent involvement of respiratory and terminal bronchioles. The inflammatory infiltrate showed a characteristic topography: the bronchiolar lumen contained neutrophils, while the peribronchiolar infiltrate was constituted of an inner layer of lymphocytes and an outer layer of histiocytes. Most of the histiocytes were foamy macrophages, which formed “nodules” distributed especially in the wall of respiratory bronchioles, in the surrounding interalveolar septa and around the blood and lymphatic vessels. In some areas, the acute inflammatory infiltrate destroyed the bronchiolar epithelium and extended to the peribronchiolar spaces, with distortion of the alveolar structure and formation of microabscesses. A severe peribronchial and peribronchiolar fibrosis was also seen (Figure 2A-F). Gram and Grocott stains were negative for bacterial and fungal microorganisms. Immunohistochemical stains showed that CD79a + and CD20+ B-cells were almost absent (Figure 3A-B) and that there was a heterogeneous population of CD4+ and CD8+ T-cells in the lymphocytic infiltrate (Figure 3C-D). The histiocytes expressed CD68, and there was a discrete number of CD1a + cells. There were no signs of GLILD. Upon examination of the histological slides at polarized light microscope, there were no traces of extrapulmonary fibers. The final diagnosis, according also to the radiological and clinical findings and to the clinical history of the patient, was acute necrotizing and chronic fibrosing panbronchitis and panbronchiolitis.

## Discussion

To the best of our knowledge, the case reported herein is the first case described in the literature of panbronchiolitis in a patient affected by CVID. The development of granulomatous disease or auto-immunity in CVID are two of the most problematic clinical complications [14]. Although granulomas can be found in many organs, lungs are the most frequent and relevant location [15]. In some patients an intense lymphoid infiltrate is associated with granulomas, realizing what has been termed GLILD [13]. DPB is a rare disease characterized by chronic inflammation of respiratory bronchioles and sinobronchial infection [8]. Inflammatory nodular lesions around the bronchioles occur diffusely throughout the lungs. Histopathologically, the nodules consist of lymphocytes and foamy macrophages around respiratory bronchioles. Interstitial accumulations of foamy macrophages in the wall of respiratory bronchioles and in the surrounding interalveolar septa represent peculiar histological features of DPB. The bronchiolar lumen contains neutrophils. Lymphoid follicles along the airways are also frequently noted. The inflammatory infiltrate destroys the bronchiolar epithelium and extends to peribronchiolar spaces. The cause of diffuse panbronchiolitis is still unknown, although it is reasonable to think that environmental factors and genetic susceptibility contribute to the development of the disease. Immunogenetic studies revealed a strong association with HLA-B54 in Japanese, whereas an association with HLA-A11 was reported in Koreans [8]. Neutrophils and T-lymphocytes, particularly CD8+ cells, together with the cytokines interleukin (IL)-8 and macrophage inflammatory protein-1 are believed to play key roles in the development of DPB. Previous reports have suggested that the accumulation of neutrophils and IL-8 secretion in the airway lumen plays an important role in the pathogenesis of the disease [16]. It is possible to speculate that neutrophil chemotactic factors at the site of inflammation and upregulation of adhesion molecules in the circulation can promote the entry of neutrophils into the airways. The accumulation of activated neutrophils in the airways may damage epithelial cells, by releasing oxidative and proteolytic products, and promote the development of extensive bronchiectases [6]. The pathogenetic significance of bronchus-associated lymphoid tissue (BALT) is less clear, as well as the observed increase, in absolute number, of CD3+ cells. In addition, a significant increase both in the percentage and in the absolute number of activated CD8+ cells has been observed [17]. The number of CD4+ cells is also increased [18]. A marked increases of the number of dendritic cells (DCs), as in our case, is found in both the bronchiolar epithelium and in submucosal tissues of patients with DPB. The observed increase and activation of DCs in DPB may support an enhanced immune response around the bronchioles through the promotion of antigen presentation [19]. The increase of foamy cells is considered a consequence of an abnormal uptake of surfactant proteins by macrophages, caused by airway obstruction or metabolic impairment [20]. However, the interstitial location of foamy macrophages renders these causes unlikely. The hypothesis that a disorder of the immune system is implicated in the pathogenesis and the development of DPB is also supported by the association of DPB with other conditions as CF, BLS, HTLV-1 infection and rheumatoid arthritis [6]. In our patient, PCR detection of most common respiratory viruses (including HTLV-1) was negative. Our case is different from the cases of DPB previously reported in the literature because there is no lymphoid hyperplasia and the nodules of foamy macrophages are more numerous and present both in perivascular and interstitial tissues. Moreover, there are neither sign of GLILD nor of the other granulomatous lesions described in literature in patients affected by CVID. In our case, a not yet identified trigger, probably of infectious origin, caused the accumulation of activated neutrophils in the airways, leading to a sequential activation of inflammatory pathways. In our patient, the reduced B-cell immunocompetence probably played an important role in the activation of an alternative immune response, characterized by CD4+ and CD8+ T-cells and also involving activated macrophages. According to our findings, the formation of nodules of foamy macrophages does not seem to depend on lymphoid hyperplasia, which is absent in our case, but probably depends on an insufficient or altered B or T lymphocytic response. Thus, the B-lymphocytic deficiency in our DPB would have led to an increase of nodules of foamy macrophages, involving also the peribronchial lymphatic and haematic vessels.

## Conclusions

The case described herein is interesting not only because it is the first case of DPB in a patient affected by CVID reported in the literature, but also because it further supports the heterogeneity of the clinical manifestations of CVID. It seems to confirm the correlation between an immunodeficiency status and the development of DPB. It provides more information on the accumulation of nodules of foamy macrophages in DPB, but further studies are needed to better understand the pathogenesis of DPB and its relationship with immunodeficiency status. However, it should be remembered that in cases like that reported herein an interdisciplinary case evaluation is necessary to find correct diagnoses [21].

### Consent

Written informed consent was obtained from the patient for publication of this Case Report and any all accompanying images. A copy of the written consent is available for review by the Editor-in-Chief of this journal.

## Abbreviations

DPB: diffuse panbronchiolitis; COPD: chronic obstructive pulmonary disease; CVID: common variable immunodeficiency; HLA: human leukocyte antigen; CF: cystic fibrosis; BLS: bare lymphocyte syndrome; HTLV-1: human T-cell lymphotropic virus type 1; ILD: interstitial lung disease; NSIP: nonspecific interstitial pneumonia; LIP: lymphocytic interstitial pneumonia; GLILD: granulomatous lymphocytic interstitial lung disease; pO2: partial pressure of oxygen; pCO2: pressure of carbon dioxide; NYHA: New York Heart Association; FVC: forced vital capacity; FEV1: forced respiratory volume in one second; RV: residual volume; TLC: total lung capacity; HRCT: high resolution computed tomography; HE: haematoxylin and eosin; IL: interleukin; BALT: bronchus-associated lymphoid tissue; DCs: dendritic cells.

## Competing interests

The authors declare that they have no competing interests.

## Authors’ contributions

AG wrote the paper; AB performed analysis of the histological sections; DB and AF made contributions to acquisition of clinical data; MAGMB and MGM carried out the immunoassays; PR contributed his expertise in the field and fruitful discussion; DS coordinated the work and gave final approval of the version. All authors read and approved the final manuscript.
